# Changing trends in the prevalence of *H*. *pylori* infection in Japan (1908–2003): a systematic review and meta-regression analysis of 170,752 individuals

**DOI:** 10.1038/s41598-017-15490-7

**Published:** 2017-11-14

**Authors:** Chaochen Wang, Takeshi Nishiyama, Shogo Kikuchi, Manami Inoue, Norie Sawada, Shoichiro Tsugane, Yingsong Lin

**Affiliations:** 1 Department of Public Health, Aichi Medical University School of Medicine, Nagakute, Aichi Japan; 2 Medical Statistics, London School of Hygiene and Tropical Medicine, London, United Kingdom; 3 Epidemiology and Prevention Group, Center for Public Health Sciences, National Cancer Center, Tokyo, Japan

## Abstract

Changing trends in the prevalence of *H*. *pylori* infection in the general population over time are thought to be the main driving force behind the declining gastric cancer mortality in Japan. However, whether the prevalence of *H*. *pylori* infection itself shows a birth-cohort pattern needs to be corroborated. We performed a systematic review of studies that reported the prevalence of *H*. *pylori* infection among Japanese individuals. Meta-regression was conducted in the framework of a generalized additive mixed model (GAMM) to account for heterogeneity in the prevalence of *H*. *pylori* infection as a function of birth year. The prevalence of *H*. *pylori* infection confirmed a clear birth cohort pattern: the predicted prevalence (%, 95% CI) was 60.9 (56.3–65.4), 65.9 (63.9–67.9), 67.4 (66.0–68.7), 64.1 (63.1–65.1), 59.1 (58.2–60.0), 49.1 (49.0–49.2), 34.9 (34.0–35.8), 24.6 (23.5–25.8), 15.6 (14.0–17.3), and 6.6 (4.8–8.9) among those who were born in the year 1910, 1920, 1930, 1940, 1950, 1960, 1970, 1980, 1990, and 2000, respectively. The present study demonstrated a clear birth-cohort pattern of *H*. *pylori* infection in the Japanese population. The decreased prevalence of *H*. *pylori* infection in successive generations should be weighed in future gastric cancer control programs.

## Introduction

*Helicobacter pylori* (*H*. *pylori*), a gram-negative bacterium that colonizes the human stomach, has been evolving with humans for tens of thousands of years. Substantial evidence supports a central role for *H*. *pylori* in the pathogenesis of upper gastrointestinal diseases, including peptic ulcer and non-cardia gastric cancer^1^. Unlike other developed countries, gastric cancer burden remains high in Japan, where it is the second leading cause of cancer deaths, accounting for annual deaths of approximately 50,000^2^. The reason for the lingering high gastric cancer incidence is manifold, but a high prevalence of *H*. *pylori* infection, reportedly as high as 80% among Japanese adults over 40 years old in a 1982 study by Asaka *et al*.^3^, appears to be the major contributor. Currently approximately 40% of the Japanese adult population are estimated to be infected with *H*. *pylori* ^4^.

Numerous epidemiological studies in Japan have reported the prevalence of *H*. *pylori* infection in various time points and age groups. These findings have shown that the prevalence of *H*. *pylori* infection increases with age^5^. This phenomenon is presumably due to a birth-cohort effect, because almost all *H*. *pylori* infection is acquired prior to the age of five, and because the environment during early childhood, such as water supply system, socioeconomic status, household living environment and hygiene habits, is closely associated with *H*. *pylori* infection^6^. Given these unique characteristics of *H*. *pylori*, the prevalence by birth year would be a valuable indicator that can reflect the time trends of *H*. *pylori* infection.

Previous studies conducted in the Western population have suggested that gastric cancer, gastric ulcer and duodenal ulcer, the three main *H*. *pylori*-related diseases, exhibit a similar birth cohort pattern, with lower rates observed in subsequent generations^7^. A decline in the prevalence of *H*. *pylori* infection in the general population is thought to be the major driving force behind this common pattern, since potent risk factors other than *H*. *pylori* have not been identified. Nevertheless, whether *H*. *pylori* prevalence itself shows a birth-cohort pattern remains to be corroborated. To our knowledge, there is no systematic review or meta-analysis consolidating the data on the prevalence of *H*. *pylori* infection from studies involving Japanese individuals. Therefore, we systematically reviewed the existing literature that presented estimates of the prevalence of *H*. *pylori* infection in the Japanese population. We aimed to derive a robust prevalence estimate of *H*. *pylori* infection by birth year, and to explore the factors that may be associated with between-study variations in *H*. *pylori* infection in our meta-regression analysis. These findings will help to inform gastric cancer screening policies.

## Methods

The PRISMA statement for preferred reporting of systematic reviews and meta-analyses was used as a guide to conduct this study.

### Data sources and Search strategy

Using the databases of PubMed and EMBASE, we performed a systematic review of the published studies on the prevalence of *H*. *pylori* infection in the Japanese population. The search on PubMed was limited to those studies that were conducted in human and to those that were published from inception to 30 June, 2016 with the following search terms: (“Helicobacter” [Mesh] OR “Helicobacter pylori” [title/abstract]) AND (“Prevalence” [Mesh] OR “prevalence” [title/abstract] OR “infection rate”) AND (“Japan” [Mesh] OR “Japan” [title/abstract] OR “Japanese” [title/abstract]). Similar strategies were applied in searching published studies in Embase. The search terms used in EMBASE were as follows: (“prevalence”/exp OR “prevalence”: ab, ti OR “infection rate”/exp OR “infection rate”: ab, ti) AND (“Japan”/exp OR “Japan”: ab, ti OR “Japanese”: ab, ti) AND (“helicobacter”/exp OR “helicobacter pylori”: ab, ti) AND (humans)/lim. To supplement electronic database searches, we also scrutinised the reference lists, and searched for unpublished data by contacting the head of known ongoing study projects in Japan.

### Study selection

**Figure 1:**
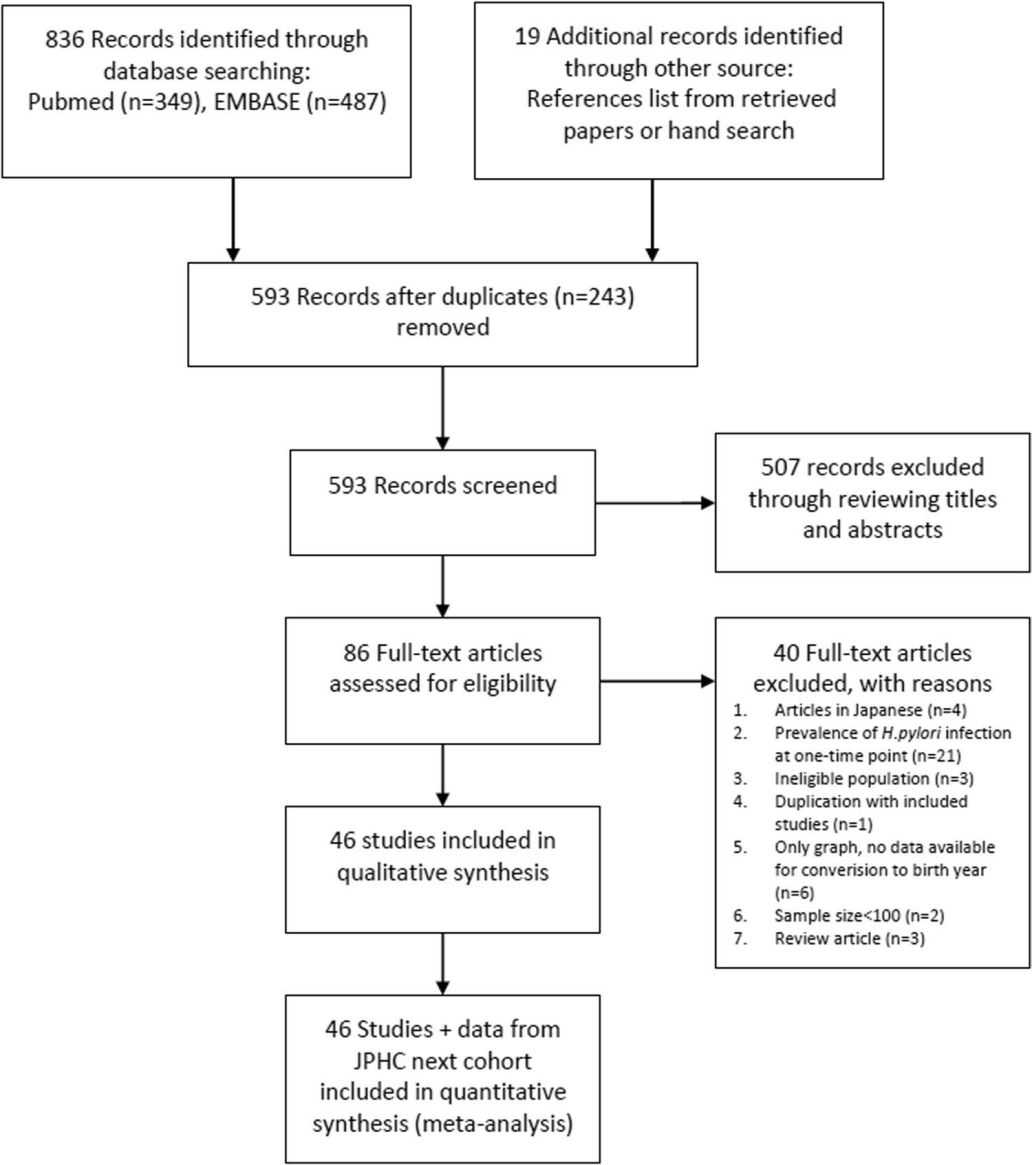
PRISMA flow chart of study selection.

After excluding the duplicate literature from the two databases, we applied the following exclusion criteria: sample size less than 100; no information on time periods during which the study was conducted; review articles; studies published in languages other than English; reports on prevalence without stratifying subjects into different age groups; patients with symptomatic digestive diseases including peptic ulcer, gastric cancer and gastric MALT lymphoma. Studies were eligible for inclusion if they were cross-sectional, case-control (only data in the control groups were extracted), or cohort studies that reported the prevalence and numbers of *H*. *pylori* infection in defined age groups (that is, age of those from whom samples were taken were specified or studies took place in population groups of a known age); or if they reported on the prevalence in any screening setting (such as community-based or hospital-based). We also included baseline data for *H*. *pylori* prevalence among 42,831 individuals who participated in the JPHC next cohort, the details of which can be accessed at the website (http://epi.ncc.go.jp/jphcnext/about/index.html). A PRISMA 2009 Flow Diagram for study selection is presented in Fig. 1.

### Data extraction and quality assessment

Two authors (LY and WC) independently searched and reviewed titles and abstracts identified by the literature search to select eligible studies. Citations identified by either reviewer were selected for full-text review. The same two authors then independently assessed the full-text articles, using predefined inclusion and exclusion criteria. Discrepancies were resolved by discussion and, if necessary, by the decision of a third author (KS). We extracted the prevalence by birth year from studies if such data were available in the original articles. And if such data were not available, we estimated birth year based on age groups and the year when the studies were conducted. The risk-of-bias assessment of all included studies was independently performed by two authors (LY and WC) using the Joanna Briggs Institute Prevalence Critical Appraisal Tool, in which 10 criteria are used to evaluate the methodological quality of studies that report prevalence data^8^. The results of risk-of-bias assessment were summarized in the Supplementary Table 1.

### Statistical analysis

Based on age groups reported in the original studies and the year when the studies were conducted, we converted them to birth years. For four studies which did not report the year of research, publication year was used instead to calculate the birth year^9–12^. For the analysis, we extracted data for the prevalence of *H*. *pylori* infection by birth year from each study: a total of 300 data points from 47 studies. In synthesizing the study results, we conducted a meta-regression to account for heterogeneity in the prevalence of *H*. *pylori* infection between studies using a logit link (logistic model).

The pre-specified explanatory variables included in the meta-regression were as follows: study ID, birth year, population source (community-based or clinical-based), diagnostic testing (serological test, or others; others include: urinary assay, salivary assay, stool antigen test, ^13^C-urea breath test and gastric biopsy), types of ELISA kits for measuring *H*. *pylori* positivity (antigen derived from domestic or foreign strains), and data collection period (prior to the year 2000, or later than 2000), with study ID as a random effect and other variables as fixed effects. Community samples came from nonclinical, population-based case-control or cross-sectional studies, and clinic-based samples included participants who were outpatients or underwent health check-ups in the clinical facilities. The year 2000 was chosen as a cutoff because the Japanese national health insurance scheme has covered *H*. *pylori* eradication for treating peptic ever since.

Since we have little prior justification for assuming a linear relationship between logit (the prevalence of *H*. *pylori* infection) and birth year, we used a penalized cubic spline to model the prevalence as a function of birth year in the framework of generalized additive mixed model (GAMM) implemented in the mgcv package in R^13^.In this analysis, we weighted observations by the inverse of the sum of the within-study variance and the residual between-study variance using the meta package^14^ in R. For all tests, *P* $<$ 0.05 was considered statistically significant.

Subsequently, we performed sensitivity analyses stratified by study qualities (good or poor) which were defined according to the results of risk-of-bias diagnosis (studies that met higher or equal to 7 out of 10 criteria were defined as good quality, and the rest were defined as poor) or stratified by the year of research conducted (earlier or later than 2000), or by excluding children data points (n = 57) due to the concern that the accuracy of test kits in children has not been fully elucidated.

## Results

The screening process is detailed in Fig. 1. Of the 86 full-text articles we reviewed, 46 met the inclusion criteria^4,5,9–12,15–54^. Collectively these citations included in the present study spanned birth years starting in 1908 and ending in 2003. Table 1 presents the characteristics for each study. Collectively, we successfully included 170,752 adults in the meta-regression analysis. Most of the studies were cross-sectional studies and were conducted in health screening, outpatient, or community settings.

**Table 1:** Characteristics of studies addressing the prevalence of *H*. *pylori* infection in Japanese.

Study ID	Reference	Data collection period	Participants	Setting	Adults or Children	Random Sampling	N	Mean Age [range]	Specimen type	Measurement kit	Antigen from domestic or foreign strains	Tested (n)	Positive (n)	Prevalence (%)
1	Fukao *et al*.^9^	NA	Healthy blood donors	Community	Children & adults	No	1815	[16–64]	Serum	ELISA kit (QUIDEL Corp., San Diego)	Foreign	1815	949	53.3
2	Replogle *et al*.^39^	1980–1993	Patients screened for Hepatitis B virus at Tokyo University Hospital	Clinical- or Hospital-based	Children & adults	No	1494	[0–94]	Serum	NA	NA	1207	470	38.9
3	Kumagai *et al*.^28^	1986, 1994	Participants of a cohort study	General population	Children & adults	No	641, 549	[6–80]	Serum	GAP-IgG test (Biomerica, Newport Beach, CA)	Foreign	641, 549	510, 370	49.6, 67.4
4	Youn *et al*.^54^	1993	Patients without gastrointestinal symptoms	Clinical- or Hospital-based	Children & adults	No	580	[2–6], [7–19], [20-]	Serum	Immunoblot assay	NA	100, 260, 100	20, 108, NA	20, 40 > 75
5	Kikuchi *et al*.^26^	1996	Public service workers	Community	Adults	No	5000	[19–69]	Serum	Pilika Plate G Helicobacter, II (Biomerica Ltd., Newport Beach, CA)	Foreign	4361	1330	30.5
6	Fujisawa *et al*.^21^	1974, 1984, 1994	Participants in a health screening program	Community	Children & adults	Yes	1015	Median 35.6, [0–89]	Serum	GAP-IgG test (Biomerica, Newport Beach, CA)	Foreign	1015	426	42.0
7	Yang *et al*.^53^	1996	Employees of a manufacturing plant	Community	Adults	No	598	[20–59]	Serum	GAP-IgG test (Biomerica, Newport Beach, CA)	Foreign	545	216	39.6
8	Shibata *et al*.^41^	1997	Residents	General population	Adults	No	1207	[30–64]	Serum	GAP-IgG, Biomerica, USA	Foreign	636	310	48.7
9	Ogihara *et al*.^36^	1989–1990	Employees of small and medium-sized textile companies	Community	Adults	No	9500	[39–65 + ]	Serum	Pilika Plate G Helicobacter, II (Biomerica Ltd, Newport, USA)	Foreign	8837	4268	48.3
10	Yamagata *et al*.^50^	1988	Participants of a cohort study	General population	Adults	No	2742	57 in men, 59 in women, [40–80 + ]	Serum	HM-CAP (Enteric Products Inc, Westbury, NY)	Foreign	2602	1721	66.1
11	Kurosawa *et al*.^29^	1995–1996	Elementary/junior high School students	Community	Children	No	610	6 and 14 years	Saliva	HELISAI kit	Foreign	610	83	13.6
12	Okuda *et al*.^38^	1998–1999	Asymptomatic children	Clinical- or Hospital-based	Children	No	484	[0–12]	Stool	Meridian Diagnositics, Cincinnati, USA	Foreign	484	31	6.4
13	Yamaji *et al*.^51^	1996–1997	Individuals attending a health screening program	Community	Adults	No	6489	48.1 [NA]	Serum	GAP-IgG test (Biomerica, Newport Beach, CA)	Foreign	5732	2695	47.0
14	Yamashita *et al*.^52^	1995–1996	Healthy Children in an out-patient clinic	Clinical- or Hospital-based	Children	No	336	[0–19]	Serum	HEL-p-test; AMRAD, Melbourne, Victoria, Australia	Foreign	336	59	17.5
15	Shibata *et al*.^5^	1993	Residents attending a health screening program	General population	Adults	No	2347	[30–79]	Serum	HM-CAP, EPI Inc., USA	Foreign	954	703	73.7
16	Fukuda *et al*.^10^	2003	Asymptomatic children	General population	Children	No	300	8	Serum	HEL-p-test; AMRAD, Melbourne, Victoria, Australia	Foreign	300	37	12.3
17	Kato *et al*.^11^	NA	Healthy Children	Clinical- or Hospital-based	Children	No	454	6.1[0–15]	Serum	HM-CAP and PP-CAP, En-teric Products, New York, NY, USA	Foreign	454	55	12.2
18	Kato *et al*. 2004	NA	Individuals with upper, gastrointestinal symptoms	Clinical- or Hospital-based	Adults	No	6578	[21–71 + ]	Serum	HM-CAP, Enteric Products, Incorporated, Stony Brook, NY, USA or a GAP lgG kit (Bio-Rad, Richmond, CA, USA).	Foreign	6578	3300	50.2
19	Nobuta *et al*.^12^	1995–1996	Asymptomatic individuals attending a health screening program	Community	Adults	No	250, 209	40.7 (Niigata), 39.1 (Okinawa)	Serum	HM-CAP	NA	250, 209	125, 88	50.0, 41.1
20	Kikuchi *et al*.^27^	1988–1990	Residents in various areas	General population	Adults	No	635	[40–79]	Serum	J-HM-CAP, Kyowa Medex Co. Ltd., Tokyo	Domestic	633	443	70.0
21	Kawade *et al*.^24^	1999–2001	Patients with dyspepsia	Clinical- or Hospital-based	Adults	No	644	82.7 [65–107]	Serum	NA	NA	644	337	52.3
22	Shimatani *et al*.^42^	1997–2003	University students attending a health check-up program	General population	Adults	No	530	23.7 [NA]	Biopsy & serum	Rapid urease test (CLO test, Ballard Medical Products, Utah, USA), and HM-CAP Enteric Products, Westbury, NY, USA)	Foreign	530	87	16.4
23	Sasazuki *et al*.^40^	1990–1992	Participants of a cohort study	General population	Adults	No	511	57.4 [NA]	Serum	E Plate “Eiken” H. pylori, Antibody, Eiken Kagaku Co. Ltd., Tokyo, Japan	Domestic	511	383	74.9
24	Fujimoto *et al*.^20^	1993, 2002	Residents attending a health screening program	General population	Adults	No	3819	[20–70 + ]	Serum	ELISA (JHM-CAP, Kyowa Medix Co.,Tokyo, Japan)	Domestic	3819	2116	55.4
25	Shiotani *et al*.^45^	2005–2006	University students	Community	Adults	Yes	777	19.5 [18–25]	Serum	E Plate test (Eiken Kagaku)	Domestic	777	114	14.7
26	Naito *et al*.^31^	2002–2003	Children from kindergarten or elementary school attending a health screening program	Community	Children	No	452	4, 7, 10	Urine	URINELISA H.pylori kit (Otsuka Pharmaceuticals, Tokyo)	Domestic	150, 150, 149, 149, 153, 153	8, 10, 7, 6, 6, 7	5.3, 6.7, 4.7, 4.0, 4.0, 4.6
Study ID	Reference	Data collection period	Participants	Setting	Adults or Children	Random Sampling	N	Mean Age [range]	Specimen type	Measurement kit	Antigen from domestic or foreign strains	Tested (n)	Positive (n)	Prevalence (%)
27	Hirai *et al*.^16^	2007	Asymptomatic adults	Clinic- or Hospital-based	Adults	No	235	[40–63]	Stool	TestMate Papid Pylori Antigen; BD Japan	Domestic	186	75	40.3
28	Mizuno *et al*.^30^	1987	Residents	General population	Adults	No	2589	[35–75 + ]	Serum	Pilika Plate G Helicobacter, II (Biomerica Ltd., Newport Beach, CA)	Foreign	2859	2147	75.1
29	Nakajima *et al*.^32^	1998, 2005	Individuals attending a health screening program	Community	Adults	No	384	[20–79]	Serum	E-plate (Eiken Chemical, Tokyo, Japan)	Domestic	384	192	50.0
30	Kawai *et al*.^25^	2003–2004	Patients undergoing routine health check-up	Clinic- or Hospital-based	Adults	No	418	39.2 [22–58]	Serum	E-plate (Eiken Chemical, Tokyo, Japan)	Domestic	418	141	33.7
31	Nakao *et al*.^33^	2001–2005	All first-visit outpatients at Aichi Cancer Center	Clinic- or Hospital-based	Adults	No	1465	[20–79]	Serum	E-plate (Eiken Chemical, Tokyo, Japan)	Domestic	1406	798	56.8
32	Akamatsu *et al*.^15^	2007–2009	Junior high school students	General population	Children	No	1232	[16–17]	Urine	RAPIRAN Otsuka Pharmaceutical Co, Tokyo, Japan	Domestic	1224	64	5.2
33	Toyoda *et al*.^47^	2004–2007	Residents	General population	Adults	No	1728	57.8 [30–89]	Serum	JHM-CAP, Kyowa, Medex Co., Ltd., Tokyo, Japan	Domestic	1540	923	59.9
34	Tamura *et al*.^46^	2008–2010	Participants of a cohort study	General population	Adults	No	5167	[35–69]	Urine	Rapiran (Otsuka Pharmaceutical Co., Ltd., Tokyo, Japan)	Domestic	5167	1881	36.4
35	Shimoyama *et al*.^43^	2005	Healthy adults attending a health screening program	General population	Adults	No	1048	[25–85]	Serum	E-plate (Eiken Chemical, Tokyo, Japan)	Domestic	1048	638	60.9
36	Urita *et al*.^48^	1999–2004	Children attending a clinic	Clinic- or Hospital-based	Children	No	838	12.4 [1–18]	Serum	NA	NA	828	101	12.1
37	Nakagawa *et al*.^17^	2005–2010	Healthy adults attending a clinic	Clinic- or Hospital-based	Adults	No	268	[20–78]	UBT	NA	NA	268	175	65.3
38	Ueda *et al*.^67^	1997–2013	Individuals attending a health screening program	Community	Adults	No	14716	NA [20 + ]	Serum/ur-ine/stool	E-plate (Eiken H.pylori antibody)	Domestic	14716	5879	39.9
39	Hirayama *et al*.^4^	2008	Employees of a large company	Community	Adults	No	21144	NA [35–79]	Serum	E-plate (Eiken Chemical Co.Ltd, Tokyo, Japan)	Domestic	21144	5822	27.5
40	Okuda *et al*. 2014	2010–2011	Participants of a population-based survey	General population	Children	No	1299, 1909	NA [0–8], NA [0–11]	Stool	TestMate Pylori Antigen EIA (Wakamoto Pharmaceutical Co. Ltd)	Domestic	688, 835	13, 15	1.9, 1.8
41	Shimoyama *et al*.^44^	2012	Healthy adults attending a health survey	General population	Adults	No	810	[40–80]	Serum/st-ool	E-plate (Eiken Chemical Co.jLtd, Tokyo, Japan; Testmate EIA (Wakamoto Pharmaceutical Co., Ltd, Kyowa Medex)	Domestic	505	224	44.4
42	Watanabe *et al*.^49^	2005–2013	All first-visit outpatients at Aichi Cancer Center	Clinic- or Hospital-based	Adults	No	4698	60.5 [20–79]	Serum	E-plate (Eiken H.pylori antibody)	Domestic	4285	1607	37.5
43	Kamada *et al*.^23^	1975–1978, 1991–1994, 2010–2013	Patients undergoing endoscopy for dyspepsia or gastric cancer screening	Clinic- or Hospital-based	Adults	No	1381	[18–70 + ]	Biopsy	Giemsa or Gimenez staining	NA	289, 787, 305	216, 417, 107	74.7, 53.0, 35.1
44	Akamatsu *et al*.^18^	2007–2013	High school students	General population	Children	No	3251	[16–17]	Urine	RAPIRAN Otsuka Pharmaceutical Co, Tokyo, Japan	Domestic	3251	136	4.2
45	Nakayama *et al*. 2016^34^	2011–2013	Junior high school students	General population	Children	No	681	NA [12–15]	Serum	E-plate (Eiken Chemicals, Tokyo, Japan)	Domestic	454	14	3.1
46	Charvat *et al*.^19^, JPHC Cohort II	1993–1994	Residents in various areas	General population	Adults	Yes	21682	[30–79]	Serum	E-plate (Eiken Chemicals, Tokyo, Japan)	Domestic	21682	14809	68.3

Abbreviation: NA, Not available; UBT, ^13^C-urea breath test.

Information on JPHC next cohort (Study ID = 45) is unpublished, details available upon request.

At first, full GAMM model with all of the aforementioned potential covariates included (Model 1) was estimated. To confirm whether Model 1 could best fit our data set, two more models were also estimated: one with covariates that showed significant effects in Model 1 (Model 2); the other one with only penalized cubic spline function of birth year and the random effect function of study ID (Model 3). Table 2 summarizes Akaike’s information criterion (AIC) and Bayesian information criterion (BIC) values for all the models. Comparison of AIC and BIC showed that the full model we proposed initially (Model 1) was the best one to fit the data (Table 2, **Model 1**, 1687.895 and 1880.004, respectively). Thus, Model 1 was believed to be appropriate to further predict the prevalence of *H*. *pylori* according to birth year in Japanese. The results of fitting for the best GAMM model (Model 1) are shown in Table 3. A borderline significant effect of diagnostic test (*P* = 0.08) is suggested, while non-significant effects of source of population, types of ELISA kit, or research year is identified.

**Table 2:** Information for tested models.

	AIC	BIC	LogLik
Model 1:			
Logit(P) = s(birth year) + r(study ID) + f(source of population) + f(diagnostic test) + f(ELISA kits) + f(research year)	1687.895	1880.004	792.0792 (df = 51.87)
Model 2:			
Logit(P) = s(birth year) + r(study ID) + f(diagnostic test)	1702.257	1889.008	−800.7071 (df = 50.42)
Model 3:			
Logit(P) = s(birth year) + r(study ID)	1702.936	1890.291	−800.8835 (df = 50.58)

Abbreviations and definitions:

AIC: Akaike’s information criterion;

BIC: Bayesian information criterion;

LogLik: Log-likelihood;

P: prevalence;

s: penalized cubic spline;

r: random effect;

f: fixed effect;

df: degree of freedom.

**Table 3:** Summary statistics from fitting meta-regression in the best model.

*Fixed effect parameter estimates*:				
Variables	OR (95% CI)			*P*
Source of population				
Community-based	1			
Clinical-based	1.12 (0.73–1.52)			0.56
Diagnostic test				
Serology	1			
Others*	0.73 (0.37–1.08)			0.08
ELISA kits				
Domestic	1			
Foreign	1.15 (0.82–1.49)			0.41
Research year				
Earlier than 2000	1			
Later than 2000	0.89 (0.59–1.19)			0.43
***Approximated significance of smooth terms:***				
	**Estimated degree of freedom**	**Reference degree of freedom**	**Chi square**	***P***
Birth year	7.3	8.1	4048	<0.00001
***Random effect parameter estimates***:				
Study ID	37.2	41.0	1881	<0.00001

*Others include: urinary assay, salivary assay, stool antigen test,^13^C-urea breath test, and gastric biopsy.

**Figure 2:**
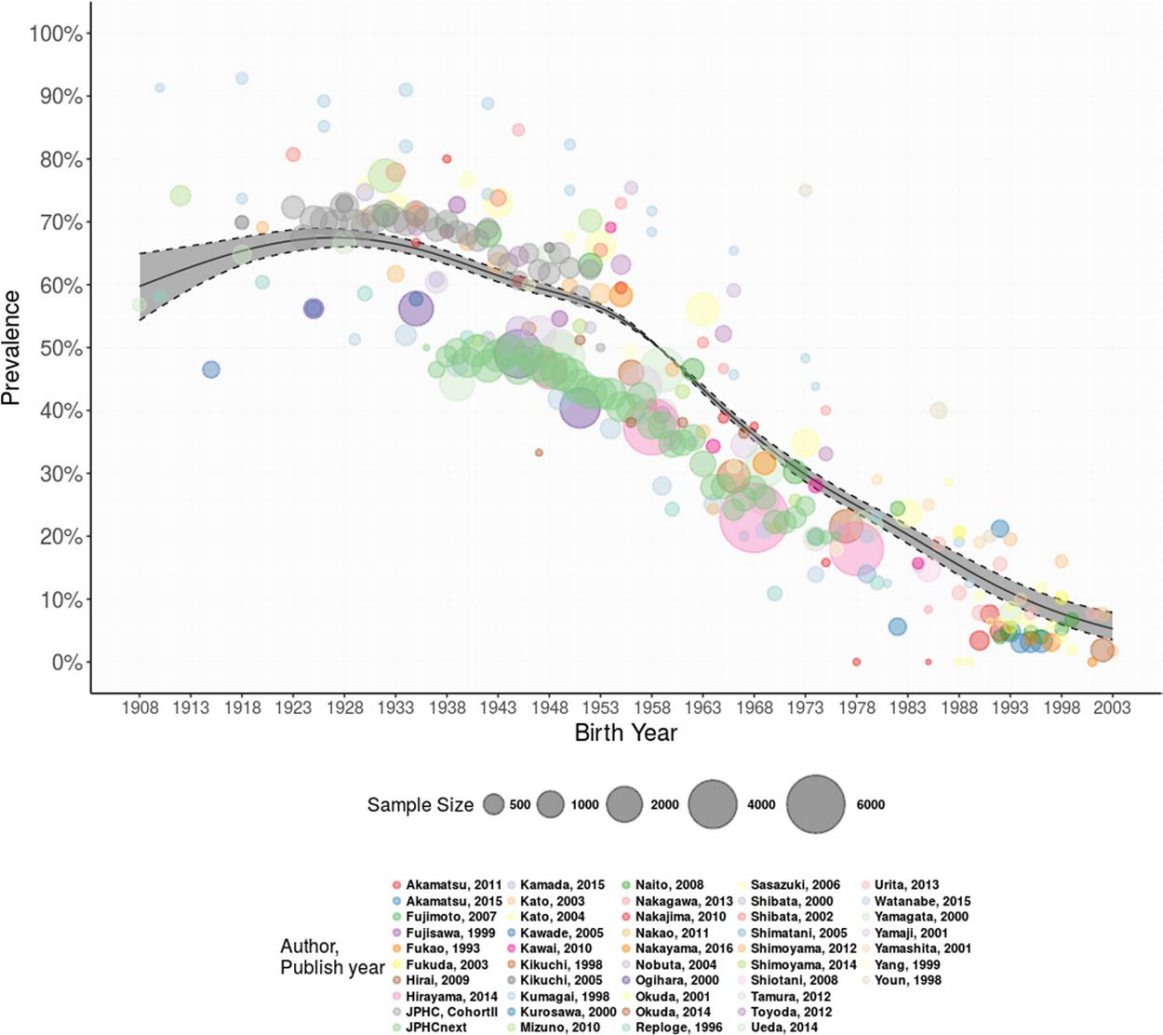
Multivariable adjusted prevalence of *H*. *pylori* infection in Japanese by birth year from year of 1908–2003.

The results also demonstrate that the smoothing trend in birth year is significant (*P* $<$ 0.00001). This decreasing trend is illustrated in Fig. 2, which depicts the smoothed curve of the relationship between *H*. *pylori* infection prevalence and birth year. The spline function estimate of prevalence indicates that the prevalence of *H*. *pylori* ranged between 50% and 70% during the first four decades (1908–1948), after which the prevalence began to decrease steadily until 2003. To be specific, the predicted prevalence (%, 95% CI) was 60.9 (56.3–65.4), 65.9 (63.9–67.9), 67.4 (66.0–68.7), 64.1 (63.1–65.1), 59.1 (58.2–60.0), 49.1 (49.0–49.2), 34.9 (34.0–35.8), 24.6 (23.5–25.8), 15.6 (14.0–17.3), and 6.6 (4.8–8.9) among those who were born in the year 1910, 1920, 1930, 1940, 1950, 1960, 1970, 1980, 1990, and 2000, respectively. The most recent cohorts, those born after 1998, appear to have a prevalence as low as less than 10% (Table 4).

**Table 4:** Predicted prevalence of *H*.*pylori* infection in Japanese population by birth year from 1908 to 2003.

Birth Year	Predicted Prevalence	95% Confidence Intervals	
1908	59.7%	54.3%	64.9%
1909	60.3%	55.3%	65.1%
1910	60.9%	56.3%	65.4%
1911	61.5%	57.3%	65.6%
1912	62.1%	58.2%	65.8%
1913	62.7%	59.1%	66.1%
1914	63.2%	60.0%	66.3%
1915	63.7%	60.8%	66.6%
1916	64.2%	61.5%	66.8%
1917	64.7%	62.2%	67.1%
1918	65.1%	62.8%	67.4%
1919	65.6%	63.4%	67.6%
1920	65.9%	63.9%	67.9%
1921	66.3%	64.4%	68.2%
1922	66.6%	64.7%	68.4%
1923	66.9%	65.1%	68.6%
1924	67.1%	65.3%	68.8%
1925	67.3%	65.6%	68.9%
1926	67.4%	65.7%	69.0%
1927	67.5%	65.9%	69.0%
1928	67.5%	66.0%	69.0%
1929	67.5%	66.0%	68.8%
1930	67.4%	66.0%	68.7%
1931	67.3%	66.0%	68.5%
1932	67.1%	65.9%	68.3%
1933	66.9%	65.7%	68.0%
1934	66.6%	65.5%	67.7%
1935	66.3%	65.2%	67.4%
1936	65.9%	64.8%	67.0%
1937	65.5%	64.4%	66.6%
1938	65.1%	64.0%	66.1%
1939	64.6%	63.5%	65.6%
1940	64.1%	63.1%	65.1%
1941	63.6%	62.5%	64.5%
1942	63.0%	62.0%	64.0%
1943	62.5%	61.5%	63.4%
1944	61.9%	60.9%	62.9%
1945	61.4%	60.4%	62.4%
1946	60.8%	59.8%	61.9%
1947	60.4%	59.3%	61.4%
1948	59.9%	58.9%	60.9%
1949	59.5%	58.5%	60.4%
1950	59.1%	58.2%	60.0%
1951	58.6%	57.8%	59.5%
1952	57.2%	56.5%	57.8%
1953	56.6%	55.9%	57.2%
1954	55.8%	55.2%	56.4%
1955	55.0%	54.5%	55.5%
1956	54.0%	53.6%	54.5%
1957	53.0%	52.6%	53.3%
1958	51.8%	51.5%	52.0%
1959	50.5%	50.4%	50.5%
1960	49.1%	49.0%	49.2%
1961	47.7%	47.4%	47.9%
1962	46.2%	45.8%	46.6%
1963	44.7%	44.2%	45.2%
1964	43.2%	42.6%	43.8%
1965	41.7%	41.0%	42.4%
1966	40.3%	39.6%	41.0%
1967	38.9%	38.1%	39.6%
1968	37.5%	36.7%	38.3%
1969	36.2%	35.4%	37.0%
1970	34.9%	34.0%	35.8%
1971	33.7%	32.7%	34.6%
1972	32.5%	31.4%	33.5%
1973	31.3%	30.2%	32.5%
1974	30.3%	29.1%	31.4%
1975	29.2%	28.0%	30.4%
1976	28.2%	27.1%	29.4%
1977	27.3%	26.1%	28.5%
1978	26.4%	25.2%	27.5%
1979	25.5%	24.4%	26.6%
1980	24.6%	23.5%	25.8%
1981	23.7%	22.5%	25.0%
1982	22.9%	21.6%	24.1%
1983	22.0%	20.7%	23.3%
1984	21.1%	19.7%	22.5%
1985	20.2%	18.8%	21.7%
1986	19.3%	17.8%	20.8%
1987	18.3%	16.9%	19.9%
1988	17.4%	15.9%	19.0%
1989	16.5%	14.9%	18.2%
1990	15.6%	14.0%	17.3%
1991	14.7%	13.0%	16.5%
1992	13.8%	12.1%	15.7%
1993	13.0%	11.3%	14.9%
1994	12.2%	10.4%	14.1%
1995	11.4%	9.7%	13.4%
1996	10.7%	8.9%	12.7%
1997	8.1%	6.4%	10.2%
1998	7.6%	5.8%	9.8%
1999	7.0%	5.3%	9.3%
2000	6.6%	4.8%	8.9%
2001	6.1%	4.3%	8.5%
2002	5.7%	3.9%	8.2%
2003	5.3%	3.5%	7.8%

Further sensitivity analyses yielded essentially similar results, which were presented as figures in supplement materials (Supplementary Figures 1–5).

## Discussion

To our knowledge, this is the first attempt to delineate the prevalence of *H*. *pylori* infection by birth year among the Japanese population based on systematic review and meta-regression analysis. Our findings suggest that *H*. *pylori* infection exhibits a birth cohort effect in Japan, with prevalence decreasing steadily in individuals born in successive years, from 59.1**%** in 1950 to 15.6**%** in 1990. In particular, the prevalence among children and adolescents is declining to very low levels, with the multivariable adjusted prevalence lower than 10% for individuals who were born after the year 1998. The multivariable adjusted prevalence of *H*. *pylori* infection seems to be lower among the older cohorts (subjects born during 1908–1918) compared with relatively younger subjects (birth year between 1923–1933) in Fig. 2. The possible reasons include potential development of atrophy or unstable estimates due to small sample sizes (the 95% CIs are much wider) among the older cohorts. Therefore, the uncertainty in prevalence estimates may exist and a cautious interpretation of results of the older cohorts is needed.

After evaluating various tools for assessing the quality of observational studies^55^, we adopted the Joanna Briggs Institute Prevalence Critical Appraisal Tool^56^, which was developed exclusively for epidemiological studies that reported on prevalence or incidence. It should be noted that even guided by such a tool, the risk-of-bias assessment is a subjective exercise. For this reason, two authors evaluated the risk-of-bias for each study independently, with disagreement resolved by either discussion or by a third author. Several concerns over methodological quality have arisen in the risk of bias assessment. First, concerning sampling strategy, most studies included in the current systematic review did not specify sampling strategy, which might have influenced the prevalence estimates owing to possible sampling bias. Second, because most studies did not explain the reasons for non-participation, it is not clear whether the study population was representative of the target population. If many individuals opted out of the survey because of illness or perceived good health, results may be an underestimate or overestimate of the real prevalence in the population. Third, serological antibody tests were used to define *H*. *pylori* infection in the majority of studies. A combination of at least two diagnostic methods is recommended to increase the validity of results, but only two studies adopted multiple tests to make a definitive diagnosis^42,44^. Fourth, controlling for two important confounders, *H*. *pylori* eradication and gastric atrophy, was not addressed in most studies. Taken together, the varied methodological approaches in the included studies and the above-mentioned limitations may have contributed to the wide variation in prevalence estimates for *H*. *pylori* infection, resulting in high between-study heterogeneity.

Based on various prevalence estimates for various age groups in included studies, a clear birth-cohort pattern emerged from our analysis. The prevalence of *H*. *pylori* infection was lower in sequential birth cohorts of Japanese born from 1908 to 2003. The results of our meta-analysis corroborated the birth-cohort pattern for *H*. *pylori* infection that was demonstrated in several included studies^4,49^, as well as a recent study exploring age, period, and cohort effects on gastric cancer mortality^57^. Moreover, our finding of a birth-cohort pattern for *H*. *pylori* infection in Japanese is similar to that documented in the United States and China, although the trajectory of decline across birth cohorts differed in these three countries^58,59^. The prevalence estimates observed for recent, younger birth cohorts in our study were comparable to those reported in the Western countries, but they were even lower when compared with those reported in China and South Korea^60,61^, two East Asian countries shouldering a similarly high gastric cancer burden. Whether the prevalence among young birth cohorts in Japan will continue to decline or it has already reached a nadir remains to be elucidated, although studies in Europe suggest that the prevalence has reached a nadir among children in recent years^62^.

Of covariates included in the meta-regression, the birth year explains much of the heterogeneity across studies. In other words, the birth year exerts a strong influence on *H*. *pylori* prevalence. Diagnostic testing (serological tests or others) might also contribute to between-study heterogeneity. Despite the sub-optimal performance characteristics of serological tests when compared with other diagnostic tests such as the urea breath test, the vast majority of included studies used serological tests to diagnose *H*. *pylori* infection because it is easy to perform and has good negative predictive value. In general, as the prevalence of an infection falls in a community, the accuracy of serological tests suffers, with an increase in the proportion of false-positive results. This also applies to *H*. *pylori* infection and this caveat should be considered when serological tests are used to diagnose *H*. *pylori* infection in a young population with a much-declined prevalence. Other potential explanations for false-positive serological test results include cross reactivity with other antigens, recent seroconversion and laboratory error. On the other hand, serological ELISA test might yield false-negative results in individuals who had serotiters in the range of 3–10 U/mL. Therefore, the observed prevalence reflected a mix of effects from both false-positive and false-negative results, making it difficult to quantify the true prevalence in the population. Because the vast majority of previous studies were limited by adopting only one diagnostic test, a combination of serological tests and other tests is necessary to increase the accuracy of the diagnosis. In addition, our meta-regression analysis indicated that differences in antigens used in ELISA did not significantly contribute to the between-study heterogeneity (odds ratio of foreign vs. domestic: 1.15, 95% CI: 0.82–1.49, *p* = 0.41). There was a concern that accuracy of kits made in Western countries may yield more intermediate results for Japanese people when compared with kits using antigens isolated from Japanese strains (for example, E-plate). However, according to our previous study^63^, when the recommended cut-off was used, there were no significant differences in diagnostic accuracy (95% CI) (domestic vs. imported: 92.5%, 90%-95% vs. 91.2%, 89%-94%, p $>$ 0.05), which is also in line with our current finding. With the predominant use of E-plate in recent years, the differences in prevalence stemming from antigen differences should not be a serious concern.

Our study has several limitations. First, including only English-language articles may lead to an over- or underestimation of the results. However, we only identified a very limited number of Japanese-language articles, which are mostly narrative reviews or conference reports. Nevertheless, no systematic bias from the use of language restriction (English-restriction) was noted in systematic review and meta-analysis^64^. In addition, another study^65^ found that English-language papers were of higher methodological quality than papers published in languages other than English. Thus, we believe that excluding studies published in Japanese language in the present study has little effect on summary estimates of prevalence of *H*. *pylori* infection. Second, because the modeling of prevalence estimates by birth cohorts across studies was used in the present meta-analysis, we were not able to assess traditional publication bias. Third, high-quality data for estimating the prevalence of *H*. *pylori* infection in the general Japanese population are limited. In addition, regarding the covariates included in the present meta-regression analysis, the data were lacking on other *H*. *pylori* infection-related factors, such as socioeconomic status, living conditions, and personal hygiene habits. These factors may have also contributed to the declining trend of *H*. *pylori* infection prevalence in Japan. Fourth, *H*. *pylori* is characterized by its genetic diversity. Its virulence factors, such as CagA and VacA, vary geographically^66^. The effect of *H*. *pylori* genetic diversity on the changes in prevalence of *H*. *pylori* infection needs further study. Finally, although study showed that serological tests could be useful for children^67^, the accuracy of this kit in children has not yet been fully elucidated. Thus, studies that included or targeted children may generate uncertain estimates. However, excluding extracted children data points (n = 57) from the complete data set did not change the results materially (Supplementary Figure 5).

In conclusion, our study demonstrated a birth-cohort pattern of *H*. *pylori* infection among the Japanese population. Given the fact that the birth-cohort pattern of *H*. *pylori* shapes the trends of gastric cancer over time, our findings help to inform screening efforts aimed at prevention and early detection of gastric cancer in Japan. The decreased prevalence of *H*. *pylori* infection in successive generations should be weighed in gastric cancer screening programs.

## Electronic supplementary material

Supplemental information

## References

[1] Cover TL, Blaser MJ (2009). Helicobacter pylori in health and disease. Gastroenterol.

[2] Inoue M (2017). Changing epidemiology of Helicobacter pylori in Japan. Gastric Cancer.

[3] Asaka M (1992). Relationship of Helicobacter pylori to serum pepsinogens in an asymptomatic Japanese population. Gastroenterol.

[4] Ueda J (2014). Prevalence of Helicobacter pylori Infection by Birth Year and Geographic Area in Japan. Helicobacter.

[5] Shibata K (2002). Relation of Helicobacter pylori infection and lifestyle to the risk of chronic atrophic gastritis: A cross-sectional study in Japan. J Epidemiol.

[6] Banatvala N (1993). The cohort effect and Helicobacter pylori. J. Infect. Dis..

[7] Sonnenberg A (2013). Review article: Historic changes of Helicobacter pylori-associated diseases. Aliment. Pharmacol. Ther..

[8] The Joanna Briggs Institute. Checklist for Prevalence Studies. *The Joanna Briggs Institute Critical Appraisal tools for use in JBI Systematic Reviews*.

[9] Fukao A (1993). Helicobacter pylori infection and chronic atrophic gastritis among Japanese blood donors: A cross-sectional study. Cancer Causes Control.

[10] Fukuda Y (2003). Impact of CagA status on serum gastrin and pepsinogen I and II concentrations in Japanese children with Helicobacter pylori infection. J. Int. Med. Res..

[11] Kato S (2003). Helicobacter pylori and TT virus prevalence in Japanese children. J. Gastroenterol..

[12] Kato M (2004). Relationship between Helicobacter pylori infection and the prevalence, site and histological type of gastric cancer. Aliment. Pharmacol. Ther..

[13] Wood, S. *Generalized Additive Models: An Introduction with R*. (CRC Press, 2006).

[14] Thompson SG, Higgins JPT (2002). How should meta-regression analyses be undertaken and interpreted?. Stat Med.

[15] Akamatsu T (2011). Introduction of an examination and treatment for helicobacter pylori infection in high school health screening. J. Gastroenterol..

[16] Hirai I (2009). Assessment of east asian-type cagA-positive helicobacter pylori using stool specimens from asymptomatic healthy japanese individuals. J Med Microbiol.

[17] Nakagawa H (2013). Association between helicobacter pylori infection detected by the (13) c-urea breath test and low serum ferritin levels among japanese adults. Helicobacter.

[18] Akamatsu T, Okamura T, Iwaya Y, Suga T (2015). Screening to Identify and Eradicate Helicobacter pylori Infection in Teenagers in Japan. Gastroenterol. Clin. North Am..

[19] Charvat H (2016). Prediction of the 10-year probability of gastric cancer occurrence in the Japanese population: The JPHC study cohort II. Int. J. Cancer.

[20] Fujimoto Y (2007). Intrafamilial transmission of Helicobacter pylori among the population of endemic areas in Japan. Helicobacter.

[21] Fujisawa T, Kumagai T, Akamatsu T, Kiyosawa K, Matsunaga Y (1999). Changes in seroepidemiological pattern of Helicobacter pylori and hepatitis A virus over the last 20 years in Japan. Am. J. Gastroenterol..

[22] Hirayama Y, Kawai T, Otaki J, Kawakami K, Harada Y (2014). Prevalence of Helicobacter pylori infection with healthy subjects in Japan. J. Gastroenterol. Hepatol..

[23] Kamada T (2015). Time Trends in Helicobacter pylori Infection and Atrophic Gastritis Over 40 Years in Japan. Helicobacter.

[24] Kawade M (2005). Prevalence of gastric cancer decreases with age in long-living elderly in Japan, possibly due to changes in Helicobacter pylori infection status. J. Gastroenterol. Hepatol..

[25] Kawai T (2010). Helicobacter pylori infection and reflux esophagitis in young and middle-aged Japanese subjects. J. Gastroenterol. Hepatol..

[26] Kikuchi S, Kurosawa M, Sakiyama T (1998). Helicobacter pylori risk associated with sibship size and family history of gastric diseases in Japanese adults. Jpn. J. Cancer Res..

[27] Kikuchi S (2005). Serum pepsinogen values and Helicobacter pylori status among control subjects of a nested case-control study in the JACC study. J Epidemiol.

[28] Kumagai T (1998). Acquisition versus loss of Helicobacter pylori infection in Japan: Results from an 8-year birth cohort study. J. Infect. Dis..

[29] Kurosawa M, Kikuchi S, Inaba Y, Ishibashi T, Kobayashi F (2000). Helicobacter pylori infection among Japanese children. J. Gastroenterol. Hepatol..

[30] Mizuno S (2010). Prescreening of a high-risk group for gastric cancer by serologically determined Helicobacter pylori infection and atrophic gastritis. Dig. Dis. Sci..

[31] Naito Y (2008). Changes in the presence of urine Helicobacter pylori antibody in Japanese children in three different age groups. Pediatr Int.

[32] Nakajima S, Nishiyama Y, Yamaoka M, Yasuoka T, Cho E (2010). Changes in the prevalence of Helicobacter pylori infection and gastrointestinal diseases in the past 17 years. J. Gastroenterol. Hepatol..

[33] Nakao M (2011). ABO genotype and the risk of gastric cancer, atrophic gastritis, and Helicobacter pylori infection. Cancer Epidemiol. Biomarkers Prev..

[34] Nakayama, Y., Lin, Y., Hongo, M., Hidaka, H. & Kikuchi, S. Helicobacter pylori infection and its related factors in junior high school students in Nagano Prefecture, Japan. *Helicobacter* **22** (2017). 10.1111/hel.12363 27785853

[35] Nobuta A (2004). Helicobacter pylori infection in two areas in Japan with different risks for gastric cancer. Aliment. Pharmacol. Ther..

[36] Ogihara A (2000). Relationship between Helicobacter pylori infection and smoking and drinking habits. J. Gastroenterol. Hepatol..

[37] Okuda M (2015). Low prevalence and incidence of Helicobacter pylori infection in children: A population-based study in Japan. Helicobacter.

[38] Okuda M (2001). Breast-feeding prevents Helicobacter pylori infection in early childhood. Pediatr Int.

[39] Replogle ML (1996). Increased risk of Helicobacter pylori associated with birth in wartime and post-war Japan. Int J Epidemiol.

[40] Sasazuki S (2006). Effect of Helicobacter pylori infection combined with CagA and pepsinogen status on gastric cancer development among Japanese men and women: A nested case-control study. Cancer Epidemiol. Biomarkers Prev..

[41] Shibata K (2000). Green tea consumption and chronic atrophic gastritis: A cross-sectional study in a green tea production village. J Epidemiol.

[42] Shimatani T (2005). Prevalence of Helicobacter pylori infection, endoscopic gastric findings and dyspeptic symptoms among a young Japanese population born in the 1970s. J. Gastroenterol. Hepatol..

[43] Shimoyama T (2012). ABC screening for gastric cancer is not applicable in a Japanese population with high prevalence of atrophic gastritis. Gastric Cancer.

[44] Shimoyama T (2014). Decrease of serum level of gastrin in healthy Japanese adults by the change of Helicobacter pylori infection. J. Gastroenterol. Hepatol..

[45] Shiotani A, Miyanishi T, Kamada T, Haruma K (2008). Helicobacter pylori infection and allergic diseases: Epidemiological study in Japanese university students. J. Gastroenterol. Hepatol..

[46] Tamura T (2012). Prevalence of Helicobacter pylori infection measured with urinary antibody in an urban area of Japan, 2008-2010. Nagoya J Med Sci.

[47] Toyoda K (2012). Serum pepsinogen and Helicobacter pylori infection–a Japanese population study. Eur. J. Clin. Microbiol. Infect. Dis..

[48] Urita Y (2013). Role of infected grandmothers in transmission of Helicobacter pylori to children in a Japanese rural town. J Paediatr Child Health.

[49] Watanabe M (2015). Declining trends in prevalence of Helicobacter pylori infection by birth-year in a Japanese population. Cancer Sci..

[50] Yamagata H (2000). Impact of Helicobacter pylori infection on gastric cancer incidence in a general Japanese population: The Hisayama study. Arch. Intern. Med..

[51] Yamaji Y (2001). Inverse background of Helicobacter pylori antibody and pepsinogen in reflux oesophagitis compared with gastric cancer: Analysis of 5732 Japanese subjects. Gut.

[52] Yamashita Y, Fujisawa T, Kimura A, Kato H (2001). Epidemiology of Helicobacter pylori infection in children: A serologic study of the Kyushu region in Japan. Pediatr Int.

[53] Yang X, Nishibayashi H, Takeshita T, Morimoto K (1999). Prevalence ofHelicobacter pylori infection in Japan: Relation to educational levels and hygienic conditions. Environ Health Prev Med.

[54] Youn HS (1998). Comparison of Helicobacter pylori infection between Fukuoka, Japan and Chinju, Korea. Helicobacter.

[55] Shamliyan T, Kane RL, Dickinson S (2010). A systematic review of tools used to assess the quality of observational studies that examine incidence or prevalence and risk factors for diseases. J Clin Epidemiol.

[56] Munn Z, Moola S, Riitano D, Lisy K (2014). The development of a critical appraisal tool for use in systematic reviews addressing questions of prevalence. Int J Health Policy Manag.

[57] Wang C, Weber A, Graham DY (2015). Age, period, and cohort effects on gastric cancer mortality. Dig. Dis. Sci..

[58] Yeh JM (2013). Contribution of H. pylori and smoking trends to US incidence of intestinal-type noncardia gastric adenocarcinoma: A microsimulation model. PLoS Med..

[59] Yeh JM, Goldie SJ, Kuntz KM, Ezzati M (2009). Effects of Helicobacter pylori infection and smoking on gastric cancer incidence in China: A population-level analysis of trends and projections. Cancer Causes Control.

[60] Lim SH (2013). Prevalence and risk factors of Helicobacter pylori infection in Korea: Nationwide multicenter study over 13 years. BMC Gastroenterol.

[61] Nagy, P., Johansson, S. & Molloy-Bland, M. Systematic review of time trends in the prevalence of Helicobacter pylori infection in China and the USA. *Gut Pathogens* **8** (2016). 10.1186/s13099-016-0091-7 PMC4791971 26981156

[62] Hoed CMden (2011). Helicobacter pylori and the birth cohort effect: Evidence for stabilized colonization rates in childhood. Helicobacter.

[63] Obata Y (2003). Diagnostic accuracy of serological kits for helicobacter pylori infection with the same assay system but different antigens in a Japanese patient population. J. Med. Microbiol..

[64] Morrison A (2012). The effect of english-language restriction on systematic review-based meta-analyses: A systematic review of empirical studies. Int J Technol Assess Health Care.

[65] Jüni P, Holenstein F, Sterne J, Bartlett C, Egger M (2002). Direction and impact of language bias in meta-analyses of controlled trials: Empirical study. Int J Epidemiol.

[66] Yamaoka Y (2010). Mechanisms of disease: Helicobacter pylori virulence factors. Nat Rev Gastroenterol Hepatol.

[67] Ueda J (2014). Diagnostic accuracy of the e-plate serum antibody test kit in detecting helicobacter pylori infection among japanese children. J Epidemiol.

