# Supplementary material for: Changing trends in the prevalence of H. pylori infection in Japan (1908–2003): a systematic review and meta-regression analysis of 170,752 individuals
Source: Sci Rep. 2017 Nov 14;7:15491. doi: 10.1038/s41598-017-15490-7 (PMC5686167; doi:10.1038/s41598-017-15490-7)

Scientific Reports

# Changing trends in the prevalence of *H. pylori* infection in Japan (1908-2003): a systematic review and meta-regression analysis of 170,752 individuals

Chaochen Wang[1], Takeshi Nishiyama[1], Shogo Kikuchi[1], Manami Inoue[2], Norie Sawada[2], Shoichiro Tsugane[2], Yingsong Lin[1,*]

1 Department of Public Health, Aichi Medical University, Nagakute, Aichi, Japan

2 Division of Cohort Consortium Research, Center for Public Health Sciences, National Cancer Center, Tokyo, Japan

Address for correspondence: Yingsong Lin, Department of Public Health Aichi Medical University School of Medicine. Tel: 81-561-62-3311, Fax: 81-561-62-5270; E-mail: linys@aichi-med-u.ac.jp

Supplementary TABLE 1. Results of risk of bias diagnosis diagnosis in 45 articles included in the systematic review.

| Study ID | authors, year | 1. Was the sample frame appropriate to address the target population? | 2. Were study participants sampled in an appropriate way? | 3. Was the sample size adequate? | 4. Were the study subjects and the setting described in detail? | 5. Was the data analysis conducted with sufficient coverage of the identified sample? | 6. Were valid methods used for the identification of the condition? | 7. Was the condition measured in a standard, reliable way for all participants? | 8. Was there appropriate statistical analysis? | 9-1 (eradication) Are all important confounding factors/subgroup/differences identified and accounted for? | 9-1 (atrophy) Are all important confounding factors/subgroup/differences identified and accounted for? | 10. Were subpopulations identified using objective criteria? |
|---|---|---|---|---|---|---|---|---|---|---|---|---|
| 1 | Fukao, 1993 | N | N | Y | Y | NA | Y | Y | Y | N | Y | Y |
| 2 | Replogle, 1996 | N | N | Y | Y | NA | Y | Unclear | Y | N | N | Y |
| 3 | Kumagai, 1998 | Unclear | Unclear | Y | Y | Y | Y | Y | Y | N | N | Y |
| 4 | Youn, 1998 | N | N | Y | Y | Unclear | Y | Y | Y | N | N | Y |
| 5 | Kikuchi, 1998 | Unclear | N | Y | Y | Y | Y | Y | Y | N | N | Y |
| 6 | Fujisawa,1999 | Unclear | Y | Y | Y | NA | Y | Y | Y | N | N | Y |
| 7 | Yang, 1999 | Unclear | N | Y | Y | Y | Y | Y | Y | N | N | Y |
| 8 | Shibata, 2000 | Y | Y | Y | Y | Y | Y | Y | Y | N | Y | Y |
| 9 | Ogihara, 2000 | Unclear | N | Y | Y | Y | Y | Unclear | Y | N | N | Y |
| 10 | Yamagata, 2000 | Y | Y | Y | Y | Y | Y | Y | Y | N | N | Y |
| 11 | Kurosawa, 2000 | Unclear | Unclear | Y | Y | Y | Y | Y | Y | N | N | Y |
| 12 | Okuda, 2001 | Y | N | Y | Y | Y | Y | Y | Y | N | N | Y |
| 13 | Yamaji, 2001 | N | N | Y | Y | Unclear | Y | Unclear | Y | Y | Y | Y |
| 14 | Yamashita, 2001 | N | N | Y | Y | Unclear | Y | Unclear | Y | N | N | Y |
| 15 | Shibata,2002 | Y | Y | Y | Y | Y | Y | Unclear | Y | N | Y | Y |
| 16 | Fukuda, 2003 | Unclear | N | Y | N | Unclear | Y | Unclear | Y | N | N | Y |
| 17 | Kato,2003 | N | N | Y | Y | Unclear | Y | Y | Y | N | N | Y |
| 18 | Kato,2004 | N | N | Y | Y | Unclear | Y | Unclear | Y | N | N | Y |
| 19 | Nobuta, 2004 | Unclear | N | Y | Y | Unclear | Y | Y | Y | N | Unclear | Y |
| 20 | Kikuchi, 2005 | Y | Y | Y | Y | Y | Y | Y | Y | N | Y | Y |
| 21 | Kawade, 2005 | N | N | Y | Y | Unclear | Y | Unclear | Y | N | N | Y |
| 22 | Shimatani, 2005 | Y | N | Y | Y | Unclear | Y | Y | Y | Y | Y | Y |
| 23 | Sasazuki, 2006 | Y | Y | Y | Y | NA | Y | Y | Y | N | Y | Y |
| 24 | Fujimoto, 2007 | Unclear | Unclear | Y | Y | Unclear | Y | Unclear | Y | N | N | Y |
| 25 | Shiotani, 2008 | Unclear | N | Y | Y | Unclear | Y | Unclear | Y | N | N | Y |
| 26 | Naito, 2008 | Unclear | N | Y | Y | Unclear | Y | Y | Y | N | N | Y |
| 27 | Hirai, 2009 | Unclear | N | Y | N | Unclear | Y | Y | Y | N | N | Y |
| 28 | Mizuno, 2010 | Y | N | Y | Y | Unclear | Y | Y | Y | Y | Y | Y |
| 29 | Nakajima, 2010 | N | N | Y | Y | Unclear | Y | Unclear | Y | N | N | Y |
| 30 | Kawai, 2010 | N | N | Y | Y | Unclear | Y | Unclear | Y | Y | Y | Y |
| 31 | Nakao, 2011 | N | N | Y | Y | Y | Y | Unclear | Y | N | Y | Y |
| 32 | Akamatsu, 2011 | Unclear | N | Y | Y | Y | Y | Y | Y | N | Y | Y |
| 33 | Toyoda, 2012 | Unclear | N | Y | Y | Unclear | Y | Y | Y | Y | Y | Y |
| 34 | Tamura, 2012 | Y | N | Y | Y | Unclear | Y | Unclear | Y | Y | N | Y |
| 35 | Shimoyama, 2012 | Unclear | N | Y | Y | Unclear | Y | Unclear | Y | N | N | Y |
| 36 | Urita, 2013 | N | N | Y | Y | Unclear | Y | Unclear | Y | N | N | Y |
| 37 | Nakagawa, 2013 | Y | N | Y | Y | Y | Y | Y | Y | N | N | Y |
| 38 | Ueda, 2014 | Unclear | N | Y | Y | Unclear | Y | Unclear | Y | Y | N | Y |
| 39 | Hirayama, 2014 | Unclear | N | Y | Y | Unclear | Y | Unclear | Y | Y | N | Y |
| 40 | Okuda, 2014 | Y | N | Y | Y | Unclear | Y | Y | Y | N | N | Y |
| 41 | Shimoyama, 2014 | Unclear | N | Y | Y | Unclear | Y | Y | Y | Y | Y | Y |
| 42 | Watanabe,2015 | N | N | Y | Y | Y | Y | Y | Y | N | N | Y |
| 43 | Kamada, 2015 | N | N | Y | Y | Unclear | Y | Y | Y | Y | Y | Y |
| 44 | Akamatsu, 2015 | Unclear | N | Y | Y | Y | Y | Y | Y | N | Y | Y |
| 45 | Nakayama, 2016 | Unclear | N | Y | Y | Y | Y | Y | Y | N | Y | Y |
| 46 | Charvat, 2016 JPHC Cohort II | Y | Y | Y | Y | Y | Y | Y | Y | N | Y | Y |

Yes, No, Unclear, Not applicable

Information on JPHC next cohort (Study ID=47) is unpublished, details available upon request.

Supplementary Figure 1. Multivariable adjusted prevalence of *H. pylori* infection in Japanese by birth year

**In studies meet higher or equal to 7 out of 10 criteria in risk of bias diagnosis**

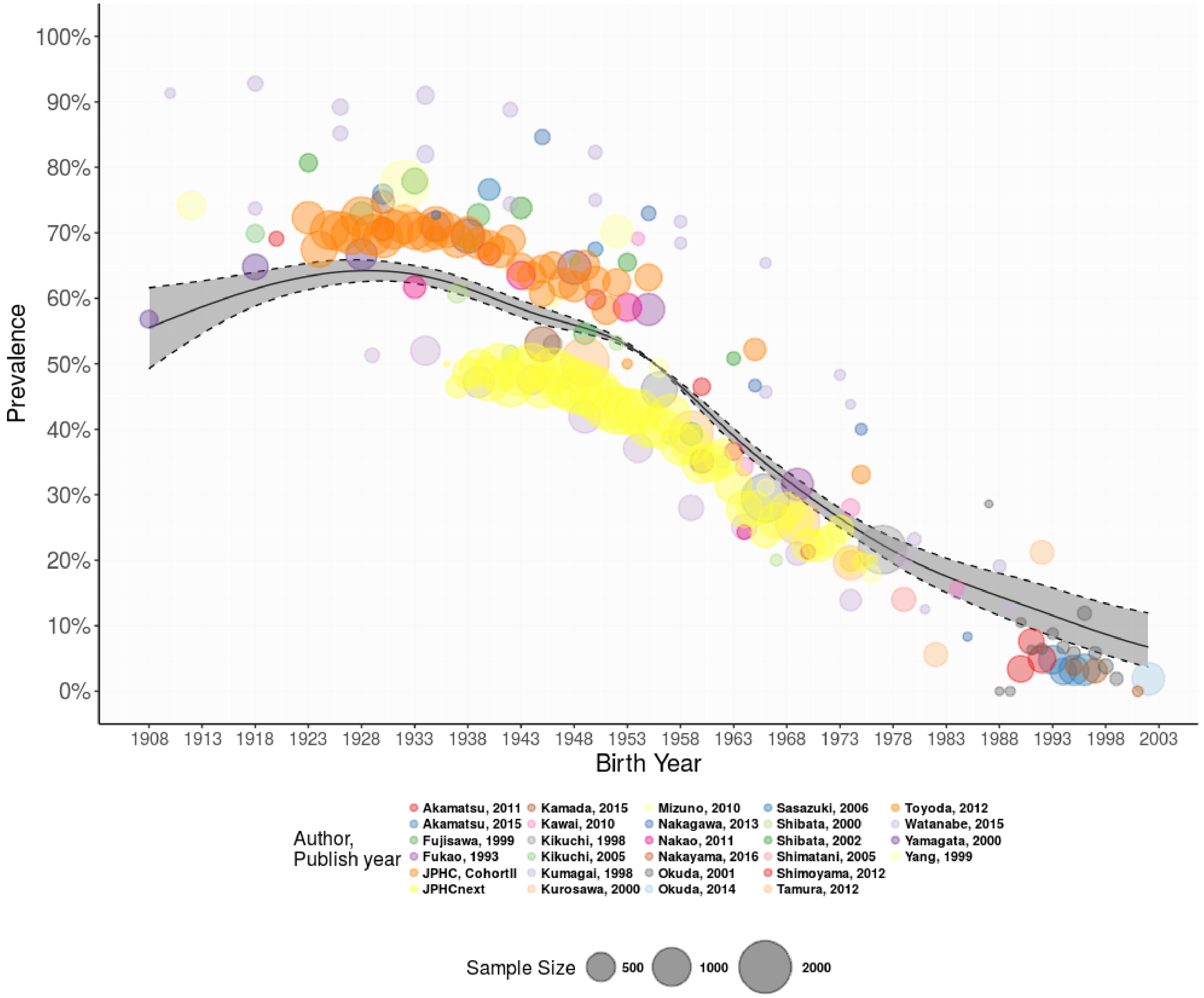

Supplementary Figure 2. Multivariable adjusted prevalence of *H. pylori* infection in Japanese by birth year

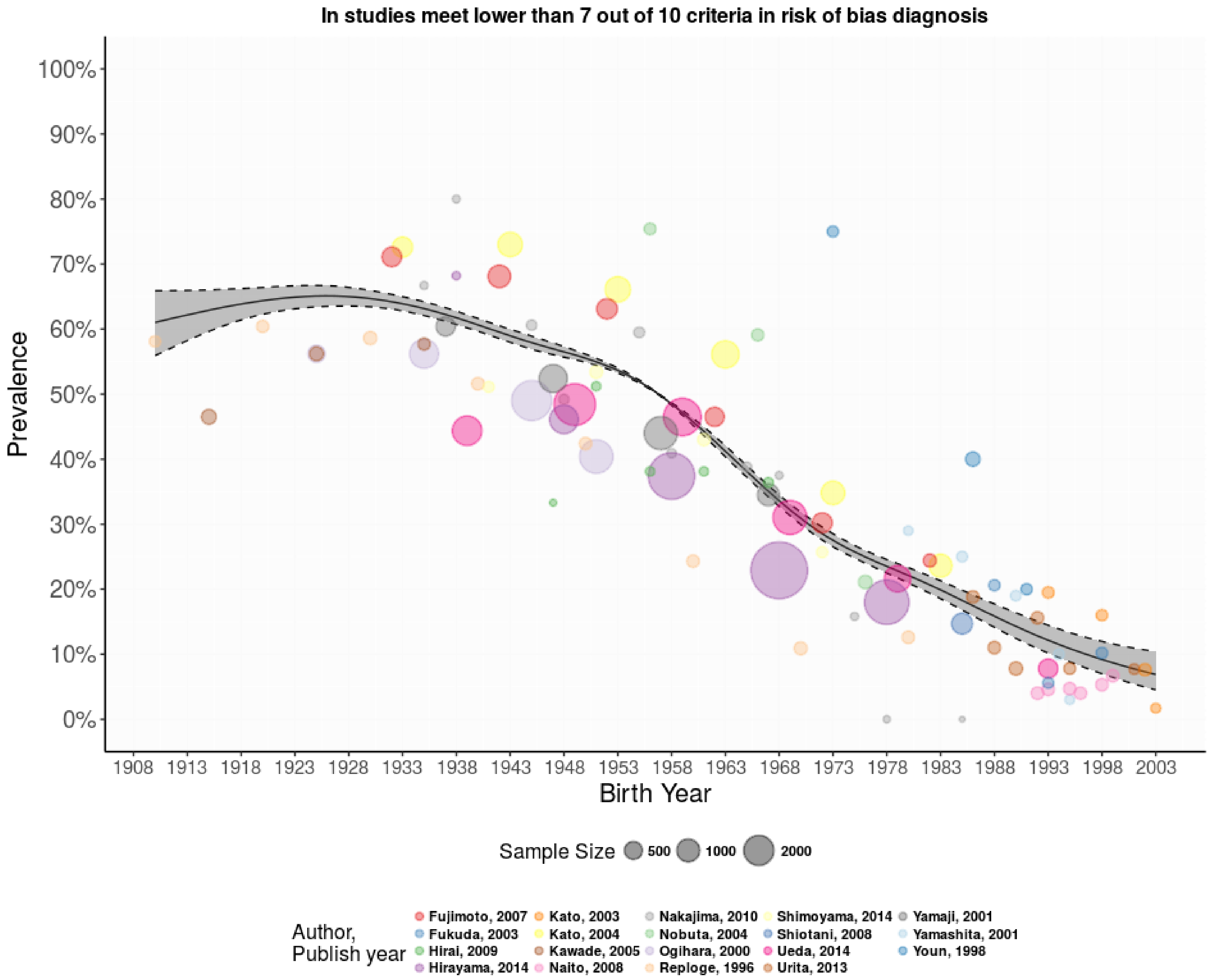

Supplementary Figure 3. Multivariable adjusted prevalence of *H. pylori* infection in Japanese by birth year

**in studies conducted earlier than 2000**

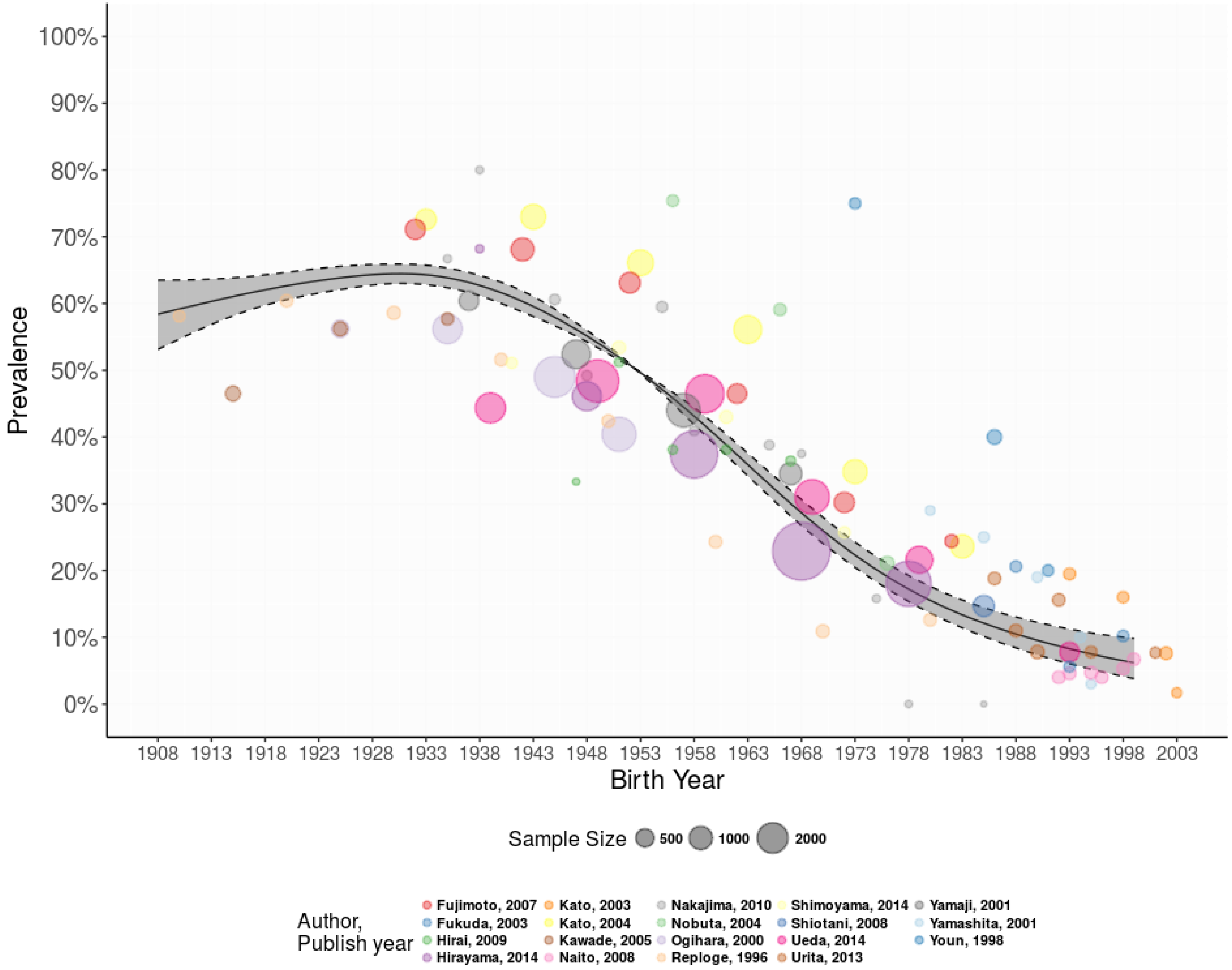

Supplementary Figure 4. Multivariable adjusted prevalence of *H. pylori* infection in Japanese by birth year

**in studies conducted later than 2000**

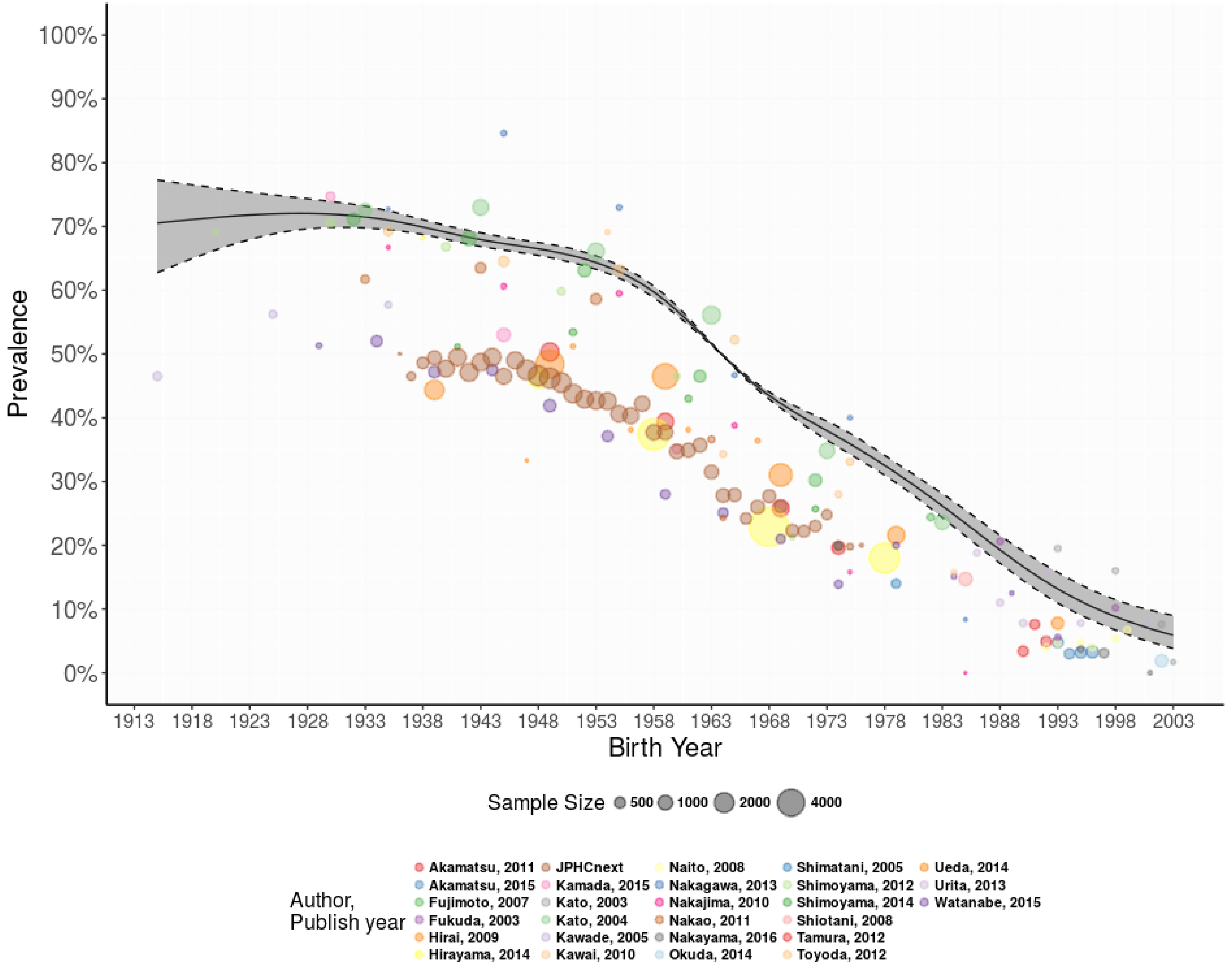

Supplementary Figure 5. Multivariable adjusted prevalence of *H. pylori* infection in Japanese adults by birth year

**from year of 1908 to 1993**

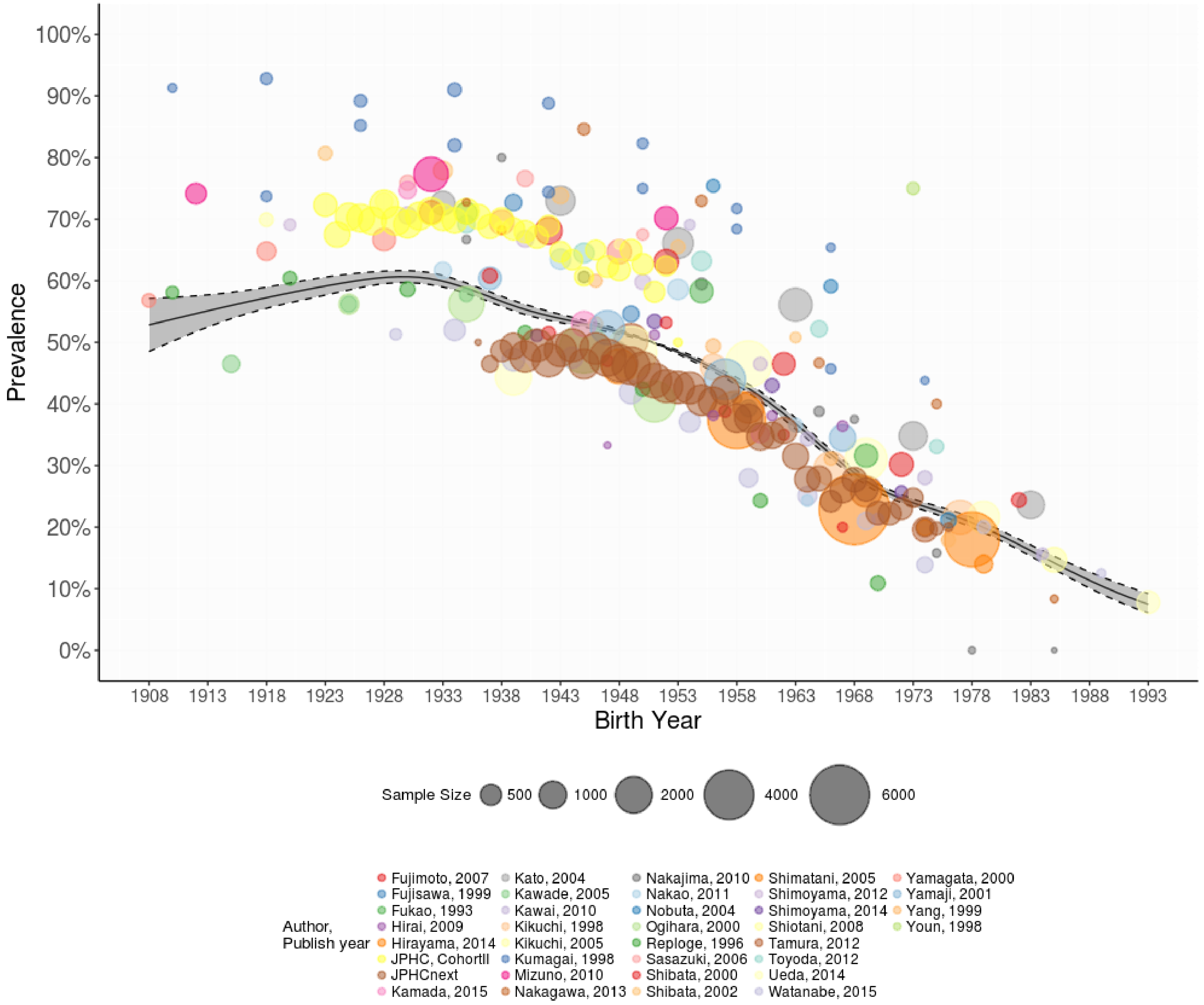

Supplement: Supplementary file 1 — Supplemental information [file 41598_2017_15490_MOESM1_ESM.pdf]
